# Supplementary material for: HIVprotI: an integrated web based platform for prediction and design of HIV proteins inhibitors
Source: J Cheminform. 2018 Mar 9;10:12. doi: 10.1186/s13321-018-0266-y (PMC5845081; doi:10.1186/s13321-018-0266-y)
Supplement: Supplementary file 2 — Additional file 2. Source code of HIVProtI web server. [file 13321_2018_266_MOESM2_ESM.zip › HIVprotI_Source-code/hivprotI/weka-3-6-6/doc/weka/classifiers/bayes/net/ParentSet.html]

ParentSet


---


|  |  |  |
| --- | --- | --- |
| **Overview** \| **Package** \| **Class** \| **Tree** \| **Deprecated** \| **Index** \| **Help** \| **Weka's home** | |  |
| **PREV CLASS**   **NEXT CLASS** | **FRAMES**    **NO FRAMES**     **All Classes** |  |
| SUMMARY: NESTED \| FIELD \| CONSTR \| METHOD | DETAIL: FIELD \| CONSTR \| METHOD |  |


---


## weka.classifiers.bayes.net Class ParentSet

```
java.lang.Object
  weka.classifiers.bayes.net.ParentSet
```

**All Implemented Interfaces:**: java.io.Serializable, RevisionHandler

---

``` public class ParentSet extends java.lang.Object implements java.io.Serializable, RevisionHandler ```

Helper class for Bayes Network classifiers. Provides datastructures to
represent a set of parents in a graph.

**Version:**
:   $Revision: 6943 $

**Author:**
:   Remco Bouckaert (rrb@xm.co.nz)

**See Also:**: Serialized Form

---

**Constructor Summary**

`ParentSet()`
  
          default constructor

`ParentSet(int nMaxNrOfParents)`
  
          constructor

`ParentSet(ParentSet other)`
  
          copy constructor


| **Method Summary** | |
| --- | --- |
| `void` | `addParent(int nParent, Instances _Instances)`             Add parent to parent set and update internals (specifically the cardinality of the parent set) |
| `void` | `addParent(int nParent, int iParent, Instances _Instances)`             Add parent to parent set at specific location and update internals (specifically the cardinality of the parent set) |
| `boolean` | `contains(int iNode)`             test if node is contained in parent set |
| `void` | `copy(ParentSet other)`             Copy makes current parents set equal to other parent set |
| `void` | `deleteLastParent(Instances _Instances)`             Delete last added parent from parent set and update internals (specifically the cardinality of the parent set) |
| `int` | `deleteParent(int nParent, Instances _Instances)`             delete node from parent set |
| `int` | `getCardinalityOfParents()`             returns cardinality of parents |
| `int` | `getFreshCardinalityOfParents(Instances _Instances)`             returns cardinality of parents after recalculation |
| `int` | `getNrOfParents()`             returns number of parents |
| `int` | `getParent(int iParent)`             returns index parent of parent specified by index |
| `int[]` | `getParents()` |
| `java.lang.String` | `getRevision()`             Returns the revision string. |
| `void` | `maxParentSetSize(int nSize)`             reserve memory for parent set |
| `void` | `SetParent(int iParent, int nNode)`             sets index parent of parent specified by index |

**Methods inherited from class java.lang.Object**

`equals, getClass, hashCode, notify, notifyAll, toString, wait, wait, wait`

**Constructor Detail**

### ParentSet

```
public ParentSet()
```

:   default constructor

---


### ParentSet

```
public ParentSet(int nMaxNrOfParents)
```

:   constructor

    **Parameters:**: `nMaxNrOfParents` - upper bound on nr of parents

---


### ParentSet

```
public ParentSet(ParentSet other)
```

:   copy constructor

    **Parameters:**: `other` - other parent set


**Method Detail**

### getParent

```
public int getParent(int iParent)
```

:   returns index parent of parent specified by index

    :   **Parameters:**: `iParent` - Index of parent **Returns:**: index of parent

---


### getParents

```
public int[] getParents()
```

---


### SetParent

```
public void SetParent(int iParent,
                      int nNode)
```

:   sets index parent of parent specified by index

    :   **Parameters:**: `iParent` - Index of parent: `nNode` - index of the node that becomes parent

---


### getNrOfParents

```
public int getNrOfParents()
```

:   returns number of parents

    :   **Returns:**: number of parents

---


### contains

```
public boolean contains(int iNode)
```

:   test if node is contained in parent set

    :   **Parameters:**: `iNode` - node to test for **Returns:**: number of parents

---


### getCardinalityOfParents

```
public int getCardinalityOfParents()
```

:   returns cardinality of parents

    :   **Returns:**: the cardinality

---


### getFreshCardinalityOfParents

```
public int getFreshCardinalityOfParents(Instances _Instances)
```

:   returns cardinality of parents after recalculation

    :   **Returns:**: the cardinality

---


### maxParentSetSize

```
public void maxParentSetSize(int nSize)
```

:   reserve memory for parent set

    :   **Parameters:**: `nSize` - maximum size of parent set to reserver memory for

---


### addParent

```
public void addParent(int nParent,
                      Instances _Instances)
```

:   Add parent to parent set and update internals (specifically the cardinality of the parent set)

    :   **Parameters:**: `nParent` - parent to add: `_Instances` - used for updating the internals

---


### addParent

```
public void addParent(int nParent,
                      int iParent,
                      Instances _Instances)
```

:   Add parent to parent set at specific location
    and update internals (specifically the cardinality of the parent set)

    :   **Parameters:**: `nParent` - parent to add: `iParent` - location to add parent in parent set: `_Instances` - used for updating the internals

---


### deleteParent

```
public int deleteParent(int nParent,
                        Instances _Instances)
```

:   delete node from parent set

    :   **Parameters:**: `nParent` - node number of the parent to delete: `_Instances` - data set **Returns:**: location of the parent in the parent set. This information can be used to restore the parent set using the addParent method.

---


### deleteLastParent

```
public void deleteLastParent(Instances _Instances)
```

:   Delete last added parent from parent set and update internals (specifically the cardinality of the parent set)

    :   **Parameters:**: `_Instances` - used for updating the internals

---


### copy

```
public void copy(ParentSet other)
```

:   Copy makes current parents set equal to other parent set

    :   **Parameters:**: `other` - : parent set to make a copy from

---


### getRevision

```
public java.lang.String getRevision()
```

:   Returns the revision string.

    :   **Specified by:**: `getRevision` in interface `RevisionHandler`
    :   **Returns:**: the revision


---


|  |  |  |
| --- | --- | --- |
| **Overview** \| **Package** \| **Class** \| **Tree** \| **Deprecated** \| **Index** \| **Help** \| **Weka's home** | |  |
| **PREV CLASS**   **NEXT CLASS** | **FRAMES**    **NO FRAMES**     **All Classes** |  |
| SUMMARY: NESTED \| FIELD \| CONSTR \| METHOD | DETAIL: FIELD \| CONSTR \| METHOD |  |


---
